# Development of a New Method for the Absolute Quantification of Selenoproteins in Chicken Serum by Heteroatom-Tagged Proteomics

**DOI:** 10.1021/acs.jafc.5c12762

**Published:** 2026-01-27

**Authors:** Belén Callejón-Leblic, Mohammed A Hachemi, Denise Cardoso, Tamara García-Barrera

**Affiliations:** † Research Center of Natural Resources, Health and the Environment (RENSMA), Department of Chemistry, Faculty of Experimental Sciences, 16743 University of Huelva, Fuerzas Armadas Ave., 21007 Huelva, Spain; ‡ Adisseo France S.A.S., 10, Place du Général de Gaulle, 92160 Antony, France

**Keywords:** chicken serum, selenoproteins, heteroatom-tagged proteomics, inductively coupled plasma, selenoprotein W, isotopic dilution

## Abstract

Selenium (Se) is an essential trace element for chickens that develops, through selenoproteins, important physiological functions. Although selenogen expression and Western blot analyses provided valuable information, there is a lack of information about the serum selenoproteome and quantitative data. In this work, we developed a new analytical method for the absolute quantification of selenoproteins and total selenometabolites in chicken serum using heteroatom-tagged proteomics, based on chromatographic separation and Se detection as a “tag” in an atomic detector. The approach was combined with bottom-up proteomics using liquid chromatography coupled to organic mass spectrometry after a tryptic digestion, and selenoprotein W was identified in chicken serum. Our results indicated that the majority of chicken serum Se is as selenoprotein P, followed by selenoprotein W, selenometabolites, glutathione peroxidase, and selenoalbumin. The approach could be a valuable tool to assess Se and selenoprotein status for the optimisation of supplementation strategies to meet the Se requirements of poultry.

## Graphical abstract

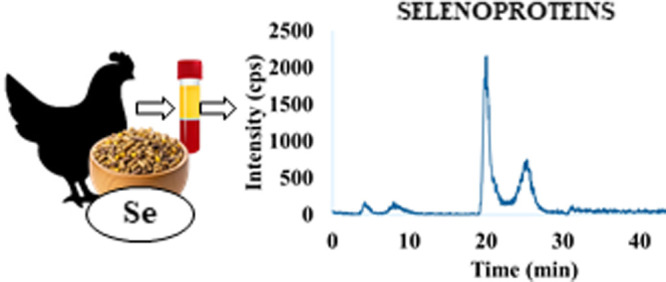

## 1 Introduction

Selenium (Se) is an essential element in poultry nutrition, playing a crucial role in antioxidant defense mechanisms.^1^ The main biological functions of Se are through selenoproteins, in which the amino acid selenocysteine (SeCys, U) is encoded by the stop codon UGA when the SeCys insertion sequence element is located in the mRNA.^2^ In chickens, 26 genes encoding different SeCys-containing proteins have been identified.^3^ Currently, there is a growing interest in the study of Se requirements and the characterization of selenoproteins in modern poultry production, driven by the positive effects on infectious diseases in poultry^4^ and the prevention of Se deficiency in consumers.^1^ Likewise, some authors have concluded that Se deficiency affects the expression of selenoprotein W (SELENOW) selenoprotein N (SEPN1), selenoprotein T (SELT), and selenoprotein K (SELK) in chicken muscle,^5^ and the expression of the gluthation peroxidases 1, 3, and 4 (GPX1, GPX3, GPX4), the thioredoxin reductases 1 and 3 (TXNRD1, and TXNRD3), the deiodinases 1 and 3 (DIO1, DIO3), Selenoprotein P (SELENOP), Selenoprotein M (SELENOM), and Selenoprotein O (SELENOO) and other selenoprotein-related enzymes such as Sep (O-phosphoserine), tRNA:Sec (selenocysteine), and tRNA synthase (SEPSECS) in chicken liver.^5^ Moreover, alterations in the expression of TXNRD1, Selenoprotein S (SELENOS), SELENOP1, and SEPSECS have been determined in chicken erythrocytes.^6^ In this context, selenogenes expression and Western blot analysis have been applied to whole blood,^7^ serum,^8,9^ neutrophils,^9^ or tissues,^7,8^ aorta vessels,^10^ and stomach^11^ in chickens. However, there is a lack of studies that focus on selenoproteins rather than selenogenes expression and that quantify them in biological chicken samples. The absolute quantification of selenoproteins in serum samples is possible by heteroatom-tagged proteomics that involves the separation of selenoproteins by high-performance liquid chromatography (HPLC), followed by the quantification of Se (used as a “tag”) by inductively coupled plasma mass spectrometry (ICP-MS).

Although the human selenoproteome comprises 25 selenoproteins (24 in mice because they do not have the human GPx6), there are only three selenoproteins present in human serum: glutathione peroxidase (GPx, ∼15–20% of total Se), selenoprotein P (SELENOP, >50% of total Se), and thioredoxin reductase (TrxR) (not quantified by ICP-MS due to the low levels). In addition, the Se-containing albumin (SeAlb, ∼15–20%) is also present in human serum. Thus, the first three selenocompounds (GPx, SELENOP, and SeAlb) account for most of Se in plasma (80–90%), and GPx and SELENOP are the most commonly used markers for the assessment of Se status in human serum.^12^

The determination of selenoproteins in chickens has been performed by the analysis of their gene expression, Western blot analysis, or the enzyme-linked immunosorbent assay (ELISA) in whole blood,^7^ serum,^8^ neutrophils,^9^ or tissues.^7,8^ In this context, these techniques have several limitations, including the inability to provide a relative protein abundance or absolute quantification. This necessitates careful optimization to ensure accurate and reproducible results. Additionally, the availability of specific and high-quality antibodies is crucial; if a suitable antibody is not available for the protein of interest, then the technique cannot be employed. Furthermore, some antibodies used in ELISA kits may not effectively distinguish between isoforms of selenoprotein P or between similar proteins. There are also sensitivity issues when the Se concentration is low, compounded by the absence of Se standards and calibrants.

However, to the best of our knowledge, only a few studies have quantified the concentration of selenoproteins in biological chicken samples. This method has been applied and validated to human serum,^12–16^ rats,^17^ or mice (García-Sevillano et al., 2014); however, there is currently no validated analytical method for the complete speciation of selenoproteins in chicken blood. This represents a significant gap in poultry research and nutritional assessment. In this study, we developed a novel analytical method utilizing heteroatom-tagged proteomics to achieve chromatographic separation of selenoproteins with the highest Se content and their absolute quantification in chicken serum. The method was combined with bottom-up proteomics for the identification of unknown Se-containing peaks using ultrahigh performance liquid chromatography coupled to quadrupole time-of-flight (UHPLC-QTOF).

## 2 Materials and Methods

### 2.1 Reagents

Reagents for tryptic digestion and purification of peptides (dithiothreitol, urea, iodoacetamide, trypsin, and NH_4_HCO_3_) were obtained from Sigma-Aldrich (Steinheim, Germany), as was ammonium acetate used for the preparation of mobile phases. Enriched ^74^Se was obtained from Cambridge Isotope Laboratories (Andover, MA). The BCR-637 human-serum-certified reference material (CRM) was purchased from the Institute for Reference Materials and Measurements (IRMM, Geel, Belgium). Water was purified with a Milli-Q Direct 8 system (Millipore, Watford, UK).

### 2.2 Instrumentation

The determinations of selenoproteins in chicken serum were carried out using an HPLC model 1260 Infinity Quaternary LC (Agilent Technologies, Tokyo, Japan) coupled to an Agilent 8800 triple quadrupole ICP-MS (ICP-QQQ-MS, Agilent Technologies, Tokyo, Japan). The serrations of selenoproteins consisted of a column switching method which connects two 5 mL HiTrap Desalting columns (SEC, 16 mm × 25 mm, GE Healthcare, Uppsala, Sweden) and a 1 mL-heparin-sepharose column (HEP-HP, 7 × 25 mm, GE Healthcare, Uppsala, Sweden) to a 1 mL-blue-sepharose column (BLU-HP, 7 × 25 mm, GE Healthcare, Uppsala, Sweden) using a six-way valve. Moreover, a T-shape unit connects the system with a Micromist nebulizer inlet (Glass Expansion, Port Melbourne, Australia) using 30 cm PEEK tubing (0.6 mm i.d.).

Lyophilization of the fractions was carried out using a freeze-dryer CRYODOS Telstar (Terrasa, Spain).

After the isolation of the chromatographic peaks and tryptic digestion, peptides were analyzed using a UHPLC Agilent 1290 Infinity with a Zorbax RRHD Rapid Resolution C18 column (50 × 2.1 mm, 18 μm) coupled to a 6550 iFunnel QTOF mass spectrometer equipped with a dual electrospray ionization (ESI) source operated in positive mode (Agilent Technologies, Tokyo, Japan).

### 2.3 Sample Collection

A pool of 50 mL of 10 chicken sera was provided by Adisseo France S.A.S. for the selenoprotein analysis. A total of 10 one-day-old Ross 308 broiler chicks were fed a single-phase basal diet (0–14 days), based on cereals (corn and wheat) and soybean meal supplemented with 0.3 mg/kg of Se (in the form of Na_2_SeO_4_). At day 14, blood samples were collected in dry sterile tubes and then centrifuged at 3500 × *g* for 15 min at room temperature. The separated serum samples were stored at −20°C for subsequent analyses. The experimental protocols and procedures were designed in compliance with the European Directive 2010/63/EU and relevant French regulations governing the care and use of experimental animals. The identification number of the institution’s approval is G-031594, and the licensing committee is the French Ministry of Higher Education and Scientific Research (#28533).

### 2.4 Heteroatom-Tagged Proteomics for Selenoproteins Quantification in Chicken Serum

The absolute quantification of the selenoproteins GPx, Se-metabolites (Se-MTB), SELENOP, and SeAlb in serum chicken was performed using a previous method applied to human serum^15^ with some modifications. This method was based on two-dimensional separation, combining affinity (AF) and size exclusion chromatography (SEC), coupled to ICP-QQQ-MS using the operational conditions described in Table S1 of the Supporting Information. Nickel cones were used for both the sampling and skimmer, with a sampling depth set at 10 mm. The RF forward power was maintained at 1550 W. Argon gas flow rates were adjusted to 15 L/min for the plasma and 1.08 L/min for the carrier gas. Se detection was carried out using high-purity oxygen (40%, >99.999%) combined with 2 mL/min of pure hydrogen gas (>95%) as the reaction gases. Isotopes monitored were ^74^Se, ^76^Se, ^77^Se, ^78^Se, and ^80^Se with a dwell time of 0.3 s per isotope. The instrumental conditions were optimized using a Tuning aqueous solution containing Li, Co, Y, and Tl at 1 μg L^–1^. Selenoproteins were separated using an online configuration that coupled two size exclusion columns (HiTrap Desalting) with two affinity chromatography columns via a six-port switching valve. The affinity columns were packed with heparin-sepharose (HEP-HP) and blue-sepharose (BLU-HP) stationary phases. SELENOP exhibited an affinity for both heparin- and blue-sepharose phases, whereas SeAlb selectively bound only to the blue-sepharose matrix.

The absolute quantification was performed by isotopic dilution analysis (IDA) using ^74^Se as the isotopic dilution standard. The SEC-AF-HPLC chromatographic setup was interfaced with the nebulizer of ICP-MS via a T connector, which also allowed the introduction of a postcolumn solution of ^74^Se for IDA. Ammonium acetate was used as mobile phases A (0.05 M, pH = 7.4) and B (1.5 M, pH = 7.4) at a flow rate of 1.3 mL min^–1^ using the following gradient of mobile phases: 100% of A from 0 to 12 min, from 12 to 12.10 min 50% of B, from 12.10 to 23 min 100% of B, and maintenance in 100% of B during 22 min. We applied 5 min to return to the initial conditions. The total acquisition time was set to 50 min. Before analysis, samples were centrifuged to eliminate suspended particulates, and 100 μL of serum was directly injected into the SEC-AF-ICP-QQQ-MS system.

The six-port valve sequence was optimized in two stages. In the initial phase (position A), mobile phase A was delivered for 12 min, allowing the elution of eGPx and low-molecular-weight selenometabolites at approximately 3 and 5 min, respectively, as these species are not retained by the affinity columns. The gradient was then shifted to mobile phase B to enable the elution of SELENOP and the unidentified compound at around 20 and 25 min, respectively. Next, the valve was switched to position B to isolate the BLUE-HP column, which specifically retains SeAlb. Immediately afterward, the valve was returned to Position A to facilitate the elution of SeAlb with mobile phase B, which appeared at approximately 30 min. The system was then equilibrated with mobile phase A for 5 min in preparation for the next injection. The signal intensity of Se isotopes was converted into mass flow chromatograms for quantitative analysis of Se species in serum, using the isotopic dilution equation and the ^7^ ^8^Se/^74^Se ratio. The peak areas obtained from these chromatograms were integrated using Origin 8.0 software (OriginLab Corporation), and final concentrations were determined by normalizing to the sample mass introduced (0.1000 g).

The methodology was validated using the BCR-637 reference material from the Institute for Reference Materials and Measurements (IRMM, Geel, Belgium), certified for the total Se content. Details on the precision and accuracy of the analytical method (Table S2), as well as the limits of detection and quantification (Table S3), are provided in the Supporting Information.

### 2.5 Identification of the Unknown Selenoprotein in Chicken Serum by UHPLC-ESI-QTOF-MS

#### 2.5.1 Isolation of the Unknown Selenoprotein from Chicken Serum

The unknown selenoprotein peak was isolated from chicken serum by using the optimized analytical performance described previously. A total of 40 fractions of the peak that eluted at 25 min were collected in a 15 mL Falcon tube for the identification of the selenoprotein by ESI-QTOF. To facilitate this process, the PEEK tube connecting the end of the column to the nebulizer of ICP-MS was detached and inserted into a Falcon tube. We collected 100 mL of peak volume that was concentrated using an N_2_ stream and lyophilized for 1 week using a lyophilizer. The samples were subjected to tryptic digestion.

#### 2.5.2 Enzymatic Digestion with Trypsin and Peptide Cleanup

Tryptic digestion was performed by reconstituting 100 mg of lyophilized sample 100 mg in 200 μL of a solution containing 8 M urea and 50 mM ammonium bicarbonate (pH 8.3), along with 5 μL of dithiothreitol (DTT, 180 mM). The mixture was incubated at 37 °C for 1 h. Subsequently, 5 μL of iodoacetamide (400 mM) was added, and the reaction was incubated again for 1 h in the dark. The samples were then diluted with 800 μL of 50 mM ammonium bicarbonate and treated with trypsin (4 μL, 0.1 mg mL^–1^), followed by 12 h incubation at room temperature for 12 h. Enzymatic activity was quenched by the addition of 100 μL of 0.1% (v/v) trifluoroacetic acid. This protocol was adapted from the method described by Kinter and Sherman (Kinter & Sherman, 2005). Following digestion, peptides were desalted, purified, and concentrated using ZipTips C18 (Millipore, Bedford, MA).

#### 2.5.3 Identification of the Unknown Peptides in Chicken Serum

A 10 μL aliquot of the tryptic digest was introduced into a Zorbax RRHD Rapid Resolution C18 column (50 × 2.1 mm, 18 μm). Peptides separation was achieved using mobile phase A (0.1% formic acid in water) and B (90% acetonitrile in water with 0.1% formic acid) under the following gradient conditions: 0 min, 3% B; 10 min, 35% B; 12 min, 90% B; 14 min, 90% B; 15 min, 3% B, at a constant flow rate of 0.5 mL min^–1^. The QTOF mass spectrometer was operated in positive ion mode with the following settings: drying gas at 14 L min^–1^, temperature at 250 °C, sheath gas temperature also at 250 °C with a gas flow rate of 11 L min^–1^, nebulizer gas at 35 psi, capillary voltage of 3500 V, mass scan range of *m*/*z* 300–1700 for MS, and range 50–1700 for MS/MS, with MS scan rate at 8 spectra/s and MS/MS at 3 spectra/s. Data acquisition and processing were performed using Agilent MassHunter Qualitative Analysis Software B.10.0. Peptide sequences corresponding to *Gallus gallus* serum selenoproteins (Table S4) were retrieved from the UniProt database (http://www.uniprot.org) and matched to experimental spectra using Agilent MassHunter Bioconfirm Software B.08.00.

## 3 Results

### 3.1 Optimization of the Heteroatom-Tagged Proteomics Method for Selenoprotein Quantification in Chicken Serum

The optimization of the heteroatom-tagged proteomics method was necessary to accurately determine selenoproteins in chicken serum. When the original method,^13,14^ primarily designed for selenoprotein determination in human serum, was applied to chicken serum, SELENOP coelutes at 20 min with another unknown selenoprotein not present in humans. As shown in Figure 1a, significant modifications were carried out to the gradient composition of the mobile phases and the flow rate to address this issue.

**Figure 1:**
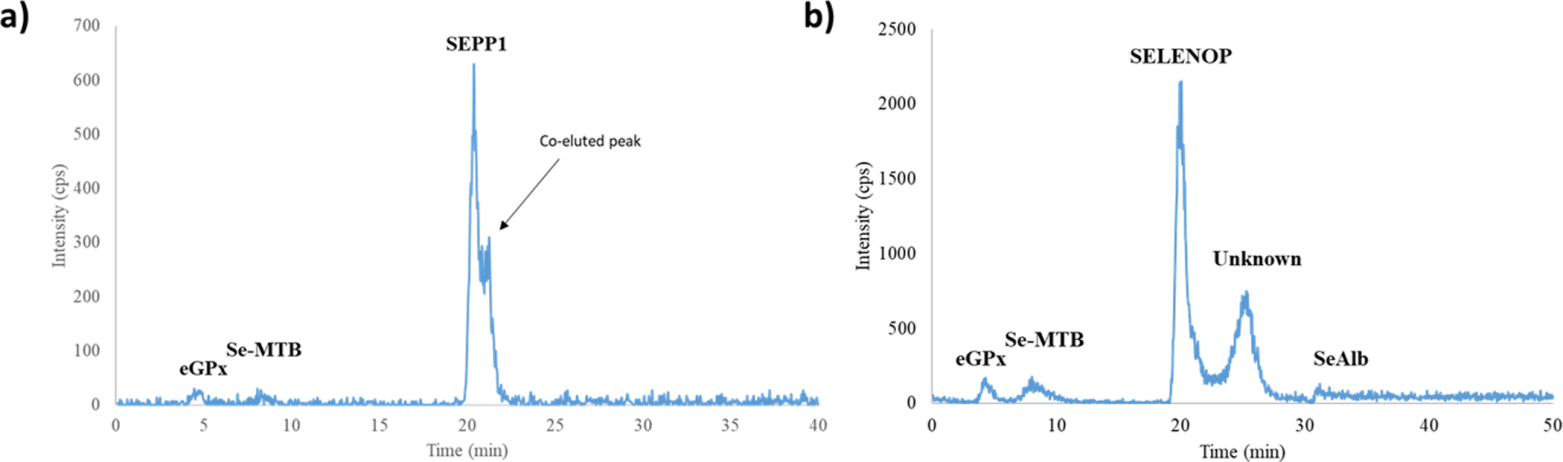
Selenoprotein profile of chicken serum using (a) the original methods and (b) the optimized method.

A comparison table (Table S5) outlines the differences between the original and optimized methods. Figure 1b illustrates the resulting chromatograms with the modification showing the optimization of the resolution between the peaks retained at 20 min (SELENOP) and the unknown peak at 25 min. It is worth noting that the retention time for eGPx and Se-metabolites remained unaffected. However, due to the increased gradient and elution time, SeAlb elutes at 31 min with the new method, which is 5 min later than in the original one.

### 3.2 Mass Spectrometric Identification of the Unknown Selenoprotein in Chicken Serum

The identification of the unknown compound chicken serum was performed after tryptic digestion of the fraction obtained through heteroatom-tagged proteomics. Peptide separation was then conducted using UHPLC-QTOF. Chromatographic profiles of the digested peptides (a) and the corresponding deconvoluted spectra (b), generated using Agilent MassHunter Qualitative Analysis Workflows B.10.00 Software, are presented in Figure 2.

**Figure 2:**
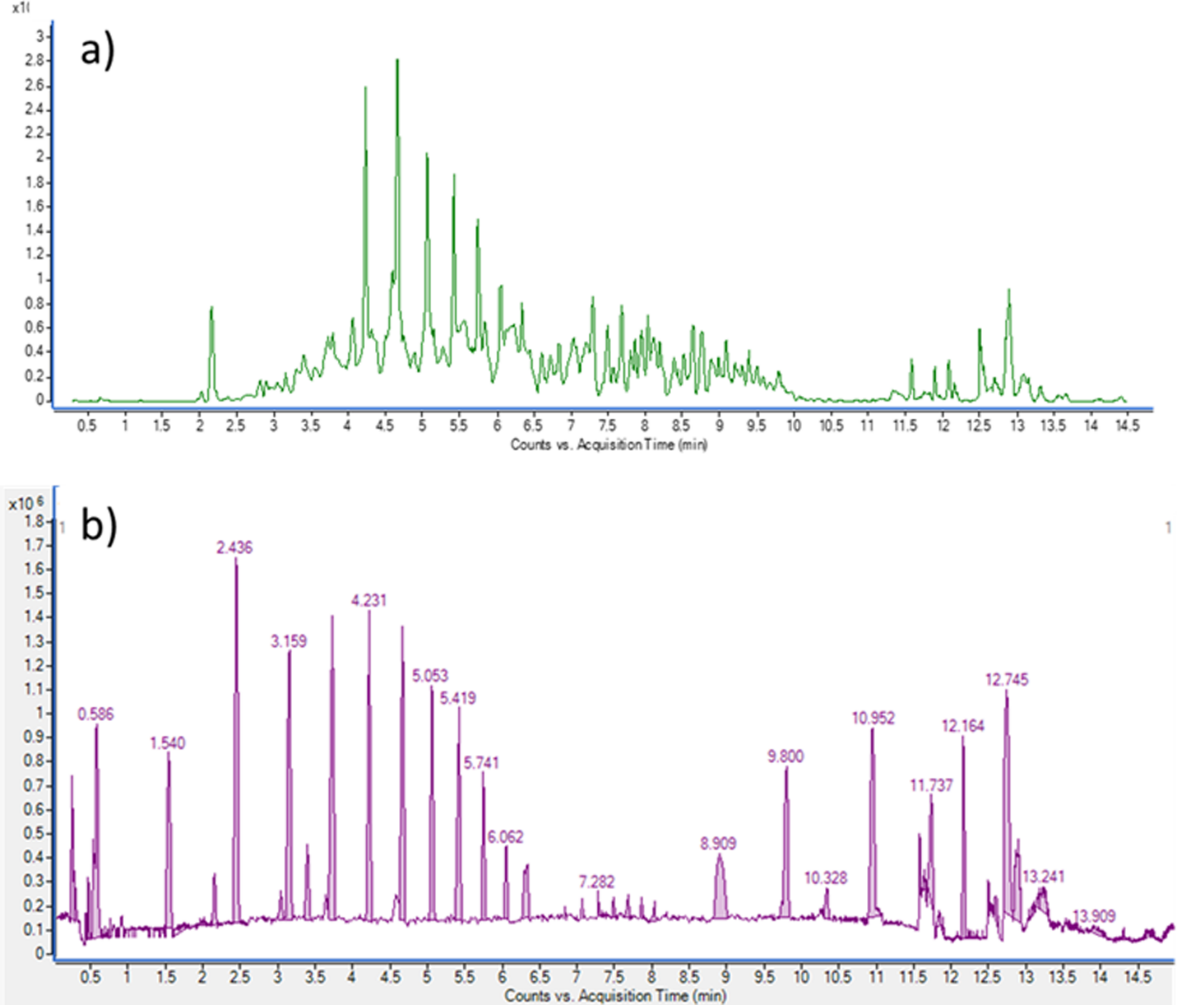
Original (a) and deconvoluted peptide (b) chromatograms of the isolated Se-containing peak from chicken serum after tryptic digestion.

After analyzing peptides in the tryptic digest, we used Agilent MassHunter Bioconfirm Software B.08.00 to match the masses with the peptide sequences of several selenoproteins using the parameters described in Table S6. Moreover, Table S7 illustrates the percentage of match sequences with the different selenoproteins. In this context, we determined the match percentage of 16 selenoprotein sequences and found that 10 sequences had a match percentage below 60% (13–59%), indicating they were not confirmed. Additionally, 6 selenoproteins had a match percentage between 60% and 75%, suggesting they are partially confirmed, while only 1 selenoprotein exceeded 88%, confirming its presence. Specifically, the partially confirmed selenoproteins are SELENOM, SELENOO, SELENOBP1, and SELENOP1. However, it is possible that a small fraction of SELENOP1 was also collected in the fraction due to its retention time being close to that of the unknown, which may explain its partial presence in the unknown fraction. However, we found that SELENOW had a sequence match of 88.24%, confirming its presence in the fraction. Figure S1 shows the sequence coverage map matched to SELENOW. In addition, Figure 3 illustrates the different peptides (a–d) matched by Bioconfimr 8.0 of SELENOW and the MS/MS spectra.

**Figure 3:**
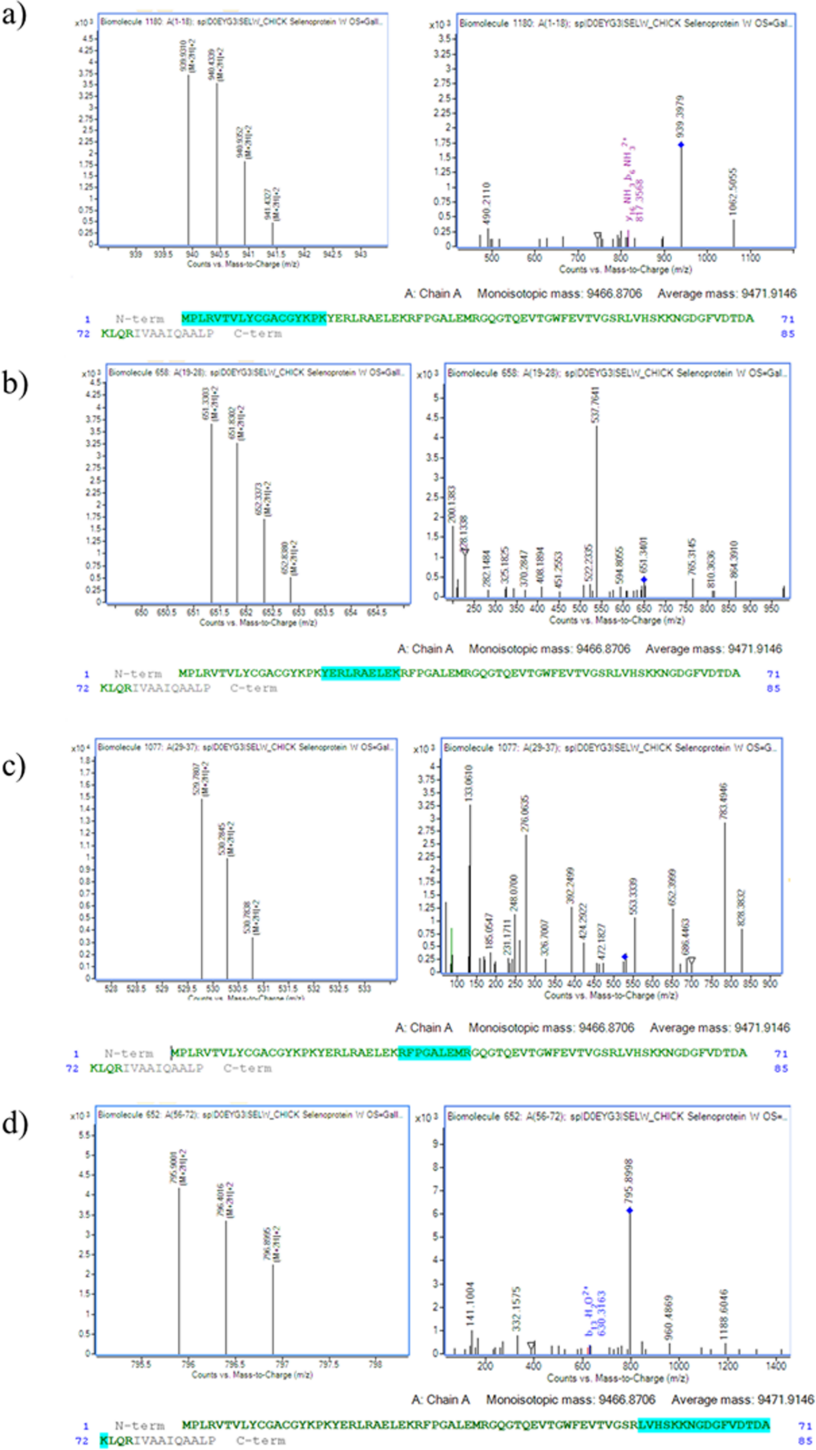
Sequence coverage map matched the SELENOW sequence determined by MassHunter Bioconfirm 8.0, Agilent Technologies.

To validate the process, bovine serum albumin (BSA) was used as a control standard for the analysis (Figure S2), obtaining a sequence match of 100% after tryptic digestion. Thus, although the unknown fraction appears to contain possibly multiple selenospecies, we can only confirm the presence of SELENOW in the fraction with a higher match.

### 3.3 Absolute Quantification of Selenoproteins in Chicken Feed Se-Supplemented Diet

As a proof of concept, the concentration of Se in the different selenoproteins was determined in 13 serum samples from chickens using the optimized methods based on IDA-SEC-AF-ICP-QQQ-MS as described in Table 1. As shown, the fraction of SELENOP corresponds to an average value of 59.2 ng of Se/ml of serum sample, representing the largest contribution to total Se across all samples. This finding is consistent with its role as the major Se-transport protein in plasma. In contrast, GPx, SMT, and SeAlb appear in much lower concentrations, typically below 5 ng of Se/mL, indicating that these species account for only a minor fraction of the circulating Se. The fraction corresponding to “SELENOW + others” constitutes the second-largest contribution (average 34.2 ng of Se/mL); it also encompasses a group of other Se-containing compounds that could not be individually resolved under the current chromatographic conditions. The relatively broad nature of this fraction explains its intermediate level of variability (SD = 5.6 ng/mL), which is higher than that observed for GPx or SeAlb but still lower than the variability associated with SELENOP.

**Table 1:** Concentration of Se in Selenoproteins from Chicken Serum^a^

	concentration of Se (ng of Se per mL of sample)					
	GPx	SMT	SELENOP	SELENOW + others	SeAlb	sum of species
sample 1	2.73	3.70	50.78	31.37	1.92	90.51
sample 2	3.01	3.80	60.84	37.77	2.34	107.76
sample 3	3.47	6.88	70.00	24.64	1.69	106.67
sample 4	3.50	2.22	63.93	30.59	1.90	102.14
sample 5	3.51	4.09	78.80	24.89	1.37	112.66
sample 6	3.58	4.66	81.42	34.68	2.46	126.81
sample 7	3.60	5.28	46.96	34.21	1.89	91.93
sample 8	3.66	1.94	53.77	33.40	1.80	94.57
sample 9	3.69	4.79	37.20	38.64	2.18	86.50
sample 10	3.80	3.57	63.31	44.27	2.48	117.44
sample 11	3.83	2.27	46.62	40.18	2.70	95.60
sample 12	3.88	6.77	46.96	35.69	1.90	95.20
sample 13	3.89	3.06	68.38	33.75	2.49	111.57
average	3.6	4.1	59.2	34.2	2.1	103.0
SD	0.3	1.6	13.4	5.6	0.4	12.0
SEM	0.1	0.4	3.7	1.5	0.1	3.3
maximum	3.9	6.9	81.4	44.3	2.7	126.8
minimum	2.7	1.9	37.2	24.6	1.4	86.5

a SMT: selenometabolites; SD: standard deviation; SEM: standard error of the mean.

Finally, we represented the percentage of selenoproteins in chicken serum (Figure S3). We observed that 57% of Se corresponds to SELENOP, followed by 33% of the SELENOW (and others), 4% of Se-metabolites, 2% of GPx, and 2% of SeAlb. To allow comparison with data from other species, we calculated the concentrations of the proteins based on the number of Se atoms in their structures. For example, SELENOP contains 13 Se atoms in the form of SeCys, whereas GPx contains only one Se atom.^18^ Accordingly, the average concentration of SELENOP in chicken serum was 2.88 mg/L, while the concentration of GPx was 1.23 mg/L. The concentration of SeAlb cannot be determined because it is not a true selenoprotein: selenium is bound nonspecifically or post-translationally to albumin, so there is no fixed stoichiometry between selenium and the protein. Similarly, the concentration of SELENOW in the serum fraction cannot be precisely determined either. This is because the fraction may contain other selenium-containing species, and the measured selenium cannot be attributed exclusively to SELENOW. As a result, any calculation of the SELENOW concentration based on total Se would be unreliable.

## 4 Discussion

The growing interest in the study of the requirements of Se in poultry has manifested the importance of developing analytical methods for the analysis of Se and selenoproteins in this field. To the best of our knowledge, this is the first time that the absolute quantification of the serum selenoproteins GPx, SELENOP, SeAlb, and Se-metabolites is determined in chicken serum by the heteroatom-tagged proteomics method using IDA, and also the first time that the SELENOW has been confirmed using UHPLC-ESI-QTOF-MS in chicken serum using this optimized methodology. Although there are also other partially confirmed selenoproteins, we have only been able to completely confirm SELENOW in the fraction. More specific studies should be carried out in the future to verify the complete confirmation of these selenoproteins in chicken serum. Moreover, as the assignment for SELENOW is based on mass and peptide identification, no commercially available antibodies are currently available to confirm this identification by Western blot or other immunological methods. Therefore, it cannot be ruled out that this signal corresponds to another low-molecular-weight selenoprotein. This limitation is important to consider in the interpretation of the data.

SELENOW is a low-molecular-weight selenoprotein (85 amino acids) localized predominantly in the cytoplasm and cell membrane^19^ that contains SeCys in position 13 of the protein sequence.^20^ Although the sequence has Se-containing amino acid U, we have not found any peptide that has the typical isotopic distribution of Se. This may be due to the different reagents used in tryptic digestion, especially DTT, a reducing agent used for the reduction of S–S bonds. We hypothesized that this amino acid was reduced or transformed. In this sense, some works have suggested that the search for the isotopic pattern of selenometabolites in spectra generated by ESI-MS is complicated and sometimes impossible due to factors such as software linked-data visualization options, large signal-to-noise ratios, compound intensity, sample complexity, and chromatographic parameters among others, also suggesting the need to use higher resolution instruments such as Orbitrap to find the pattern.^21^ Additionally, other authors have reported on the behavior of tryptic peptides containing carbamidomethyl-cysteine that are modified by cyclization, resulting in the formation of secondary structures that affect the identification of the peptide under study.^22^ Furthermore, the loss of Se from selenoproteins has also been linked to the conversion of selenocysteine to dehydroalanine in *in vitro* studies.^23^

The biological function of SELENOW remains unclear. However, some evidence suggests that it may act as an antioxidant, respond to stress, contribute to cell immunity, serve as a specific target for methylmercury, and possess a thioredoxin-like function.^24^ Although evidence of the presence of SELENOW in serum is limited, its expression is not confined solely to muscle. Some researchers have identified its presence in blood, noting that SELENOW is highly expressed in peripheral blood mononuclear cells (PBMCs) derived from multiple myeloma as well as in mature osteoclasts compared to healthy controls.^25^ Additionally, in the liver, SELENOW has been confirmed as an extensively expressed hepatic selenoprotein that plays a vital role in antioxidant functions.^26^

Our results indicated that the majority of chicken serum Se is part of SELENOP, followed by SELENOW, Se-MTB, GPx, and SeAlb. It is noteworthy that SELENOP has 13 Se atoms in chicken,^27^ while GPX has several isoforms with different Se content, whereas Se in SeAlb is in the form of Se-Met and on a random basis.^28^ Another limitation to consider in this study is that only selenite was used as the Se source during feeding. This likely explains the relatively low contribution of Se-Met-containing albumin to total Se in serum. If Se-Met had been used as the dietary Se source, then the fraction of Se incorporated into albumin would likely have been considerably higher. This is relevant for poultry nutrition, as organic Se forms such as Se-Met are more efficiently incorporated into proteins, enhancing nutritional value, whereas inorganic forms like selenite are less efficiently incorporated but may reduce the risk of selenium toxicity when used at higher levels.^1^ The main advantages of HPLC coupled to inorganic mass spectrometry (ICP-MS) versus the classical proteomic approaches based on molecular MS are sensitivity and selectivity. The reason is the indirect determination of selenoproteins using the Se signal into an atomic mass spectrometric detector, especially with instruments equipped with triple quadrupole or collision/reaction cells.^29^ The standardless and absolute quantification of selenoproteins is also possible, and the IDA contributes to important advantages, namely, (i) absence of instrumental drift; (ii) absence of matrix effects; (iii) correction with dilution or preconcentration factors are not required because isotopic ratios are used instead of monoisotopic Se signal; (iv) uncertainty only depends on the measurement of the relative abundances. Regarding Western blot analysis, only a relative quantification is possible and requires specific antibodies for each selenoprotein. The enzyme-linked immunosorbent assay (ELISA) method is simple and selective, but the antigen must be well-known (purified and isolated). On the other hand, several authors reported the enzymatic activities of several selenoproteins, but not all the selenoproteins are enzymes, and the absolute quantification is scarcely reported.^29^ The main shortcoming of HPLC-ICP-MS for selenoproteins is the analysis of biological tissues because, unlike plasma/serum that can be directly injected after filtration, there is not yet a sample preparation method with enough recovery. Thus, a choice could be polymerase chain reaction (PCR), which is very sensitive, but there is no direct connection between the alterations in gene expression and the selenoprotein levels.^30^ Therefore, with other methods, a comprehensive mass balance of Se across different selenoproteins is not possible, and they are not able to detect and quantify the full range of selenoproteins present in chicken blood.

We also determined the amount of Se in Se-MTB in the chicken serum samples. Little is known about the concentration of Se-MTB in poultry. Only the concentration of low molecular weight (LMW) of Se-compounds (inorganic Se and SeCys) has been reported in the chicken breast by SEC-ICP-MS.^31^

A new analytical approach combining heteroatom-tagged proteomics and bottom-up proteomics has been developed for the absolute quantification of selenoproteins and total Se-MTB in chicken serum.

The ICP-MS allowed a sensitive and selective determination of selenoproteins and total Se-MTB, whereas organic MS enabled the unequivocal identification of SELENOW. The method provided the first quantitative data of chicken serum selenoproteome and total Se-MTB. Results revealed that the relative Se concentration follows the order: SELENOP > SELENOW > Se-MTB > GPx > SeAlb. Our results open further research related to chicken Se metabolism, the potential impact of supplementation strategies, and the effects on chicken biology and consumer health.

## Supplementary Material



## Notes

(Table S1) Operational conditions of ICP-QQQ-MS; (Table S2) validation of selenoprotein analysis by IDA-SEC-AF-ICP-QQQ-MS using the human serum BCR-637 reference material; (Table S3) limit of detection (LOD) and quantification (LOQ) for elements and selenoproteins; (Table S4) match peptide sequences for *Gallus gallus* selenoproteins (uniprot.org); (Table S5) comparison of UHPLC conditions for determining selenoproteins in the original and new optimized method; (Table S6) MassHunter BioConfirm 8.0 parameters used for the identification of peptides; (Table S7) match percentage with protein sequence from UniProt (https://www.uniprot.org/); (Figure S1) sequence coverage map matched the SELENOW sequence determined by MassHunter Bioconfirm 8.0, Agilent Technologies; (Figure S2) bovine serum albumin peptides chromatogram and sequence coverage map of the BSA standard with a 100% match; (Figure S3) distribution of selenium among selenoproteins in chicken serum (PDF)
