# Correction to “Development of a New Method for the Absolute Quantification of Selenoproteins in Chicken Serum by Heteroatom-Tagged Proteomics”

**DOI:** 10.1021/acs.jafc.6c01559

**Published:** 2026-02-19

**Authors:** Belén Callejón-Leblic, Mohammed A Hachemi, Denise Cardoso, Tamara García-Barrera

The following sentence should be added to the Acknowledgements: Funding for the open access charge provided by Universidad de Huelva/CBUA.

