# Supplementary material for: Development of a New Method for the Absolute Quantification of Selenoproteins in Chicken Serum by Heteroatom-Tagged Proteomics
Source: J Agric Food Chem. 2026 Jan 27;74(4):4036–44. doi: 10.1021/acs.jafc.5c12762 (PMC12879916; doi:10.1021/acs.jafc.5c12762)

Supplementary Material

Development of a new method for the absolute quantification of selenoproteins in chicken serum by heteroatom-tagged proteomics

Belén Callejón-Leblic[a], Mohammed A Hachemi[b], Denise Cardoso[b], Tamara García-Barrera[a]*

[a] *Research Center of Natural Resources, Health and the Environment (RENSMA). Department of Chemistry, Faculty of Experimental Sciences, University of Huelva, Fuerzas Armadas Ave., 21007, Huelva, Spain.*

[b] *Adisseo France S.A.S., 10, Place du Général de Gaulle, 92160 Antony, France*

Corresponding author: *tamara@dqcm.uhu.es.

Table S1. Operational Conditions of ICP-QQQ-MS

**Operational Conditions**	
Nebulizer	MicroMist
Sampling and skimmer cones	Nickel
RF Power	1500 W
Plasma gas flow rate	15 L min^{-1}
Carrier gas flow rate	0.65 L min^{-1}
Auxiliary gas flow	1 L min^{-1}
Sampling depth	10 mm
He flow	4.5 ml min^{-1}
H2 flow	2 mil min^{-1}
O2 flow	40%
Dwell time	0.3 per isotope
Torch	Shield (with long life platinum shield plate)
OctP Bias	-18.0 V
OctP RF	190 V
Energy Discrimination	-7.0 V
Isotopes monitored	^{74}Se, ^{76}Se, ^{78}Se, ^{80}Se and ^{82}Se
Replicates	5

Table S2.Validation of selenoprotein analysis by IDA-SEC-AF-ICP-QQQ-MS using de human serum BCR-637 reference material.

**Human serum BCR-637 reference material (ng of Se per ml of sample)**					
**n=5**	**eGPx**	**SMT**	**SELENOP**	**SeAlb**	**Total sum**
Average	14.55	2.49	53.44	12.70	80.70
RSD (%)	4.63	6.16	4.88	3.82	3.86
Accuracy (%)	3	-	10.93	2.27	0.37
Certified value	15 ± 4*	-	60 ± 7*	13 ± 4*	81± 7
*Values reported by P. Jitaru et al. doi: 10.1007/s00216-008-2043-7. doi: 10.1016/j.aca.2009.10.037.					

Table S3. Limit of detection (LOD) and quantification (LOQ) for elements and selenoproteins. Limits of detection (LODs) and quantification (LOQs) were calculated with the data generated in the linearity studies as LOD = a + 3 Sy/x and LOQ = a + 10 Sy/x, where “a” is the origin ordinate and “Sy/x” the random errors in the values for the slope and intercept.

**Selenoproteins (ng ml^{-1})**	**LOD**	**LOQ**
eGPx	0.5	1.67
Selenometabolites	0.5	1.67
SELENOP	0.5	1.67
SeAlb	0.5	1.67

Table S4. Match Peptides Sequences for *Gallus Gallus* selenoproteins (uniprot.org). AA: amino acids

Entry	Entry Name	Protein Names	Gene Names	Length
**A0A8V0ZNE9**	A0A8V0ZNE9_CHICK	Selenoprotein M	SELENOM	151 AA
**A0A8V1AAQ7**	A0A8V1AAQ7_CHICK	Selenoprotein K	SELENOK	95 AA
**A0A8V0YPB9**	A0A8V0YPB9_CHICK	Selenoprotein P1	SELENOP1	231 AA
**A0A8V0Y558**	A0A8V0Y558_CHICK	Selenoprotein W	SELENOW	85 AA
**A0A8V0ZYK4**	A0A8V0ZYK4_CHICK	Selenoprotein F	SELENOF	184 AA
**E1C5C5**	E1C5C5_CHICK	Selenoprotein S	SELENOS	192 AA
**A0A8V0YSL9**	A0A8V0YSL9_CHICK	Selenoprotein N	SELENON	534 AA
**A0A0H4FKR5**	A0A0H4FKR5_CHICK	Selenoprotein P2	SELENOP2	108 AA
**A0A8V0X2T6**	A0A8V0X2T6_CHICK	Selenoprotein O	SELENOO	656 AA
**A0A8V0XZ47**	A0A8V0XZ47_CHICK	Selenoprotein I	SELENOI	398 AA
**A0A8V1A5K1**	A0A8V1A5K1_CHICK	Migration and invasion enhancer 1	MIEN1	166 AA
**A0A8V0Y691**	A0A8V0Y691_CHICK	Thioredoxin reductase 1	TRXD1	496 AA
**A0A8V1A972**	A0A8V1A972_CHICK	Methanethiol oxidase	SELENBP1	517 AA
**A0A8V0Y5V5**	A0A8V0Y5V5_CHICK	O-phosphoseryl-tRNA(SEC) selenium trasnferase	SEPSECS	453 AA

Table S5. Comparison of UHPLC conditions for determining selenoproteins in the original and new optimized method.

**UHPLC Conditions**		
Sample	Human Serum	Chicken Serum
Mobile Phases	A: 0.05 mM $AcNH_4$	A: 0.05 mM $AcNH_4$
	B: 1.5 mM $AcNH_4$	B: 1.5 mM $AcNH_4$
Gradient	0 -12 min: 100% A	0 -12 min: 100% A
	12-12.10 min: 100 % B	12-12.10 min: 80 % A
	12.10 – 35 min: 100% B	12.10 – 25 min: 100% B
	35 – 35.10 min: 100% A	25 – 40 min: 100% B
	35.10 – 40 min: 100 % A	40 – 45 min: 100% A
	-	45 – 50 min: 100% A
Flow (ml/min)	1.3	0 - 12.10 min: 1.3
		12.10 -30 min: 1.8
		30-50 min: 1.3

Table S6. MassHunter BioConfirm 8.0 parameters used for the identification of peptides

Parameters	
**Mass Matching**	
MS match	± 10 ppm
MS/MS match	± 30 ppm
**Workflow**	
Mode	Protein Digest
Condition	Reduced
Sequences/Masses	D0EYG3 · SELW_CHICK from UNIPROT
Modifications and Profiles	mAb
	Phospho+Ox
	Protein Digest (Reduced +Alkylated9
	Carbamylation
	DTT
	Cys as SeCys
**Scores**	
> 80 %	Protein is Confirmed
> 60%	Protein is Partially Confirmed
< 60 %	Protein is Not Confirmed

Table S7. Match percentage with protein sequence from Uniprot (https://www.uniprot.org/)

Selenoprotein	% match	Result
SELENOI	15	Not confirmed
SELENOF	27	Not confirmed
SELENON	33	Not confirmed
SELENOP2	34	Not confirmed
SELENOT	40	Not confirmed
SELENOP1	47	Not confirmed
SELENBP1	53	Not confirmed
SELENOS	57	Not confirmed
TRXD1	59	Not confirmed
SELENOM	60	Partially confirmed
SELENOO	60	Partially confirmed
SELENBP1	61	Partially confirmed
SELENOP	61	Partially confirmed
MIEN1	72	Partially confirmed
SEPSEC	74	Partially confirmed
SELENOW	88	Confirmed

Figure S1.The sequence coverage map matched the SELENOW sequence determined by MassHunter Bioconfirm 8.0, Agilent Technologies.

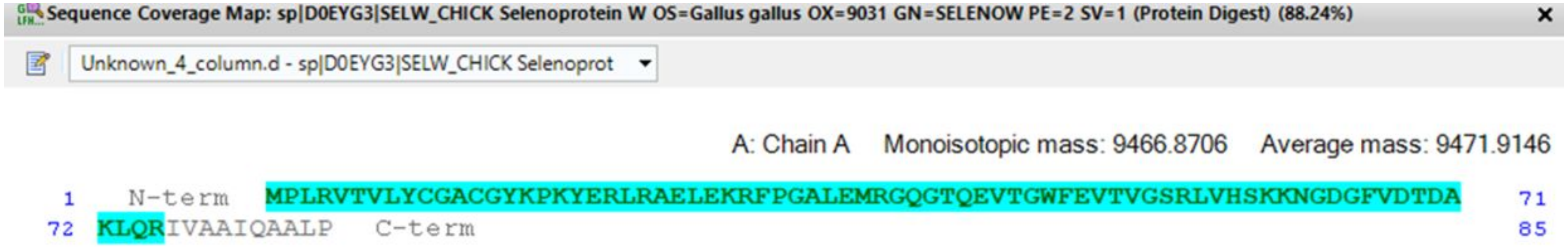

Figure S2. Bovine serum albumin peptides chromatogram and Sequence Coverage Map of the BSA standard with a 100% match.

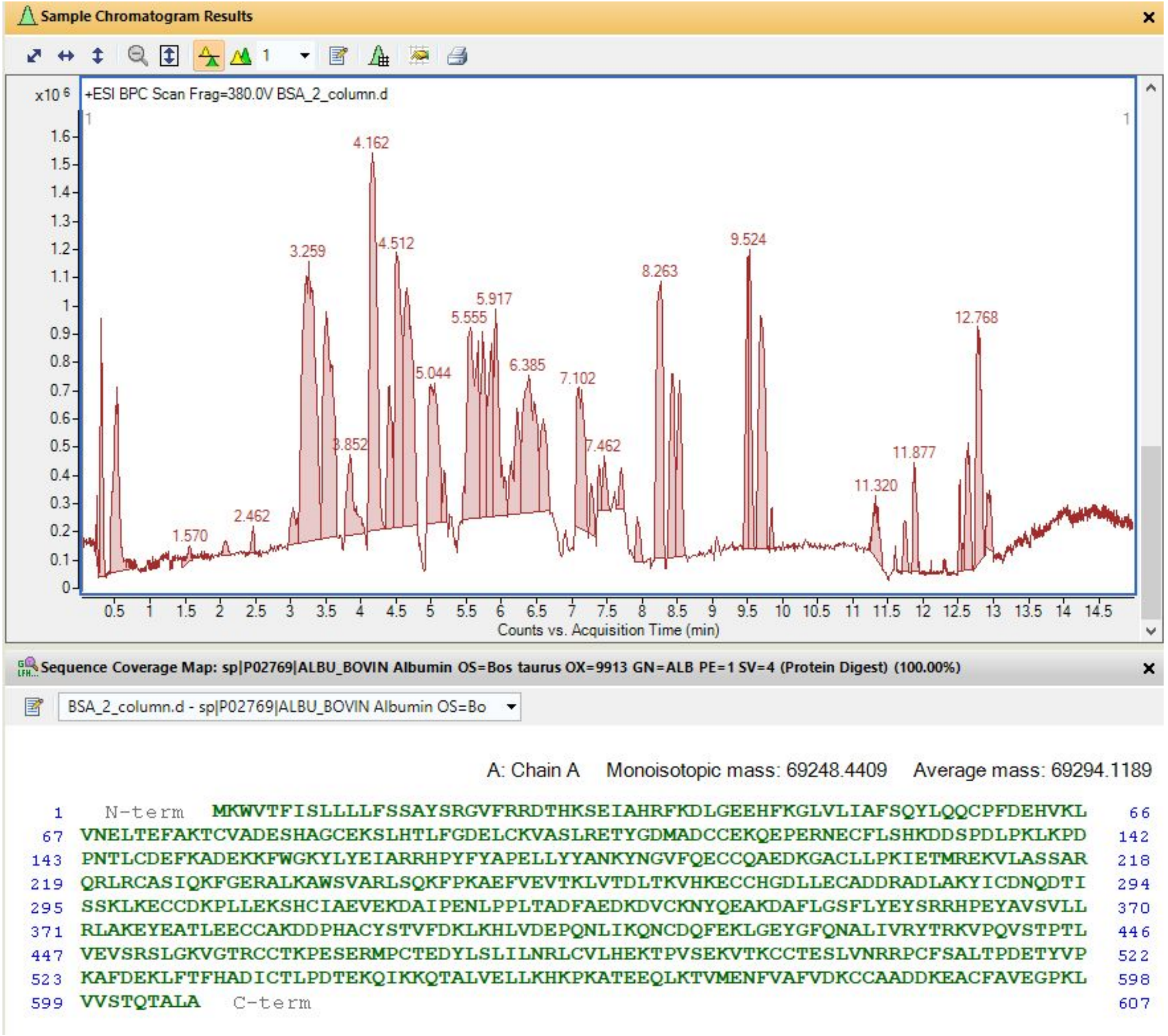

Figure S3. Distribution of selenium among selenoproteins in chicken serum.

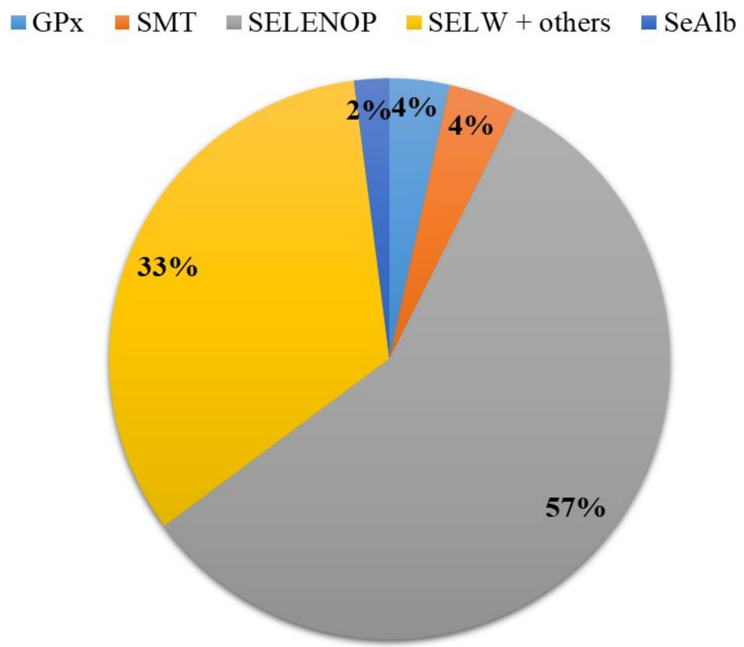

Supplement: Supplementary file 1 [file jf5c12762_si_001.pdf]
